# Improving Microalgae Research and Marketing in the European Atlantic Area: Analysis of Major Gaps and Barriers Limiting Sector Development

**DOI:** 10.3390/md19060319

**Published:** 2021-05-30

**Authors:** Judith Rumin, Raimundo Gonçalves de Oliveira Junior, Jean-Baptiste Bérard, Laurent Picot

**Affiliations:** 1 La Rochelle Université, UMRi CNRS 7266 LIENSs, Avenue Crépeau, 17042 La Rochelle, France; judith.rumin@gmail.com (J.R.); oliveira.farma.junior@gmail.com (R.G.d.O.J.); 2 Laboratoire BRM/PBA, IFREMER, Rue de l’Ile d’Yeu, 44311 Nantes, France; Jean.Baptiste.Berard@ifremer.fr

**Keywords:** cyanobacteria, microalgae, market, research, Europe, Delphi analysis

## Abstract

Microalgae and cyanobacteria represent a diverse renewable resource with significant potential for the industrial production of goods and services with high added value. However, scientific, technical/technological, legislative and market gaps and barriers still limit the growth of these markets in Europe and the number of exploited species. We conducted an in-depth survey of European microalgae researchers, experts and stakeholders to identify these limitations and to discuss strategies, recommendations and guidelines to overcome these barriers. Here, we present the findings of this study which detail the main promising markets for microalgae and cyanobacteria in the coming decades, an updated SWOT analysis of the sector, the current opportunities, limitations, risks and threats for microalgae research and market sectors in Europe, a traffic light analysis for a quick assessment of market opportunities for each microalgae sector and detailed recommendations/guidelines for overcoming the scientific, technical/technological, legislative and market gaps and barriers.

## 1. Introduction

With an estimated number of 30,000 to 72,000 species, microalgae and cyanobacteria represent a diversified renewable resource with major potential for the industrial development of products and services with high added value, in the fields of human and animal nutrition, biotechnology, cosmetics and pharmaceuticals, biomaterials and bioenergy [1,2,3,4,5,6,7]. The European Atlantic Area benefits from numerous assets in the sector as it brings together high-level research centers, technological platforms producing high-quality microalgae under controlled and standardized conditions, international research networks and innovative and competitive biotechnology companies [2,8,9]. In recent decades, the scientific community has capitalized and accumulated a more detailed understanding of microalgae biology, metabolism and chemodiversity, but only a small number of species have been screened thus far, leading to a strong limitation in the number of approved species for commercial applications. Additionally, although many studies are devoted to the use of microalgae and cyanobacteria in the fields of environment, food, chemicals, pigments, protein, feed and drugs, the market is still heavily dominated by unrefined biomass or extracts, fatty acids, proteins and carotenoids, even though microalgae can produce bioactive polysaccharides for cutting-edge pharmacological and biotechnological applications, photoprotectors, photosensitizers, antioxidants, enzymes, nanomaterials, biohydrogen, bioplastics, recombinant proteins, antibiotics and antivirals, toxins of pharmacological interest, etc. In two previous bibliometric analyses of microalgae research and markets in the world, we reported that 15 genera are mostly used for the majority of microalgae and cyanobacteria research, development and markets (representing 0.02–0.05% of the taxonomic diversity) [8,9]. These are, in descending order, *Chlorella* sp., *Scenedesmus* sp., *Chlamydomonas* sp., *Phaeodactylum* sp., *Nannochloropsis* sp., *Dunaliella* sp., *Isochrysis* sp., *Tetraselsmis* sp., *Arthrospira* sp., *Selenastrum* sp., *Botryococcus* sp., *Haematococcus* sp., *Acutodesmus* sp., *Synechocystis* sp., and *Schyzochytrium* sp. An in-depth analysis of the use and interest in each of these species in relation to research concepts, emerging research concepts, biotechnological and environmental applications, markets, countries, labs and publishing journals has been discussed in these two previous publications. As they are not part of the historical human diet, microalgae, cyanobacteria and related ingredients are covered by the "novel foods" legislation, which complicates applications for the marketing of related ingredients or purified extracts. The complete list of microalgae-related products authorized in Europe as novel foods is very restrictive and can be found at https://eur-lex.europa.eu/legal-content/EN/TXT/HTML/?uri=CELEX:02017R2470-20200308&from=FR#tocId3 (accessed on 25/05/2021). Some genera such as *Spirulina/Arthrospira* and *Chlorella* are not included in the novel food list because they are not considered as novel and regarded as GRAS (generally recognized as safe). This low number of commonly used species can even be more limited in some countries such as China where only extracts or metabolites from selected species can be sold (the “China list” includes *Chlorella emersonii*, *Chlorella minutissima*, *Chlorella vulgaris*, *Dunaliella salina*, *Euglena gracilis*, *Haslea ostrearia*, *Hematococcus pluvialis*, *Phaeodactylum tricornutum*, *Porphyridium cruentum*, *Pseudanabaena galeata*, *Skeletonema costatum*, *Spirulina maxima*, *Spirulina platensis* and *Tetraselmis suecica*). In addition to these limitations, the industrialization and commercialization of microalgae are not progressing as fast as expected because scientific, technical/technological, profitability and legislative challenges limit the research, development and marketing of related products and services [10]. Identifying these gaps and barriers was the main objective of this work as it is a critical step to unlock the innovation potential of microalgae and cyanobacteria and propose strategies, guidelines and recommendations to enhance their profitability and reach sustainable economic models. To address this issue, we performed a two-round Delphi analysis of European microalgae researchers’, experts’ and stakeholders’ opinions and recommendations. First, an in-depth survey of researchers, experts and stakeholders located in the European Atlantic Area was realized to identify the main scientific, technical/technological, legislative and commercial gaps limiting the development of the European microalgae sector. In a second step, the findings of the survey were discussed with European microalgae experts in a workshop to highlight consensus responses and to propose recommendations and guidelines to overcome these gaps and obstacles. Based on this advanced analysis, we proposed conclusions and recommendations/guidelines to facilitate research development, technology transfer, industrial development and transfer to markets. An updated SWOT analysis of the sector was carried out, and a traffic light analysis of market opportunities for each microalgae sector was proposed, allowing a quick overview of the most promising sectors of activity. The main conclusions of this study indicate the following:

(i) From a scientific point of view, the genome sequencing of microalgae species and strains remains limited, genomic data reliably interpreted by experts are scarce and the most recent genetic manipulation techniques are still marginally used and mastered on microalgae and cyanobacteria. Although they have been documented for many years, inter- and intra-species interactions in microalgae cultures are poorly studied and generate an important risk of contamination difficult to prevent when upscaling cultures from bench to industrial scales. It is necessary to improve the knowledge of the biomass composition to validate proofs of concept for microalgae products, identify minor metabolites of high interest and understand the metabolic and ecophysiological levers that enable their production to be optimized. Expertise in large-scale production and access to decision support tools for the production of microalgae and related compounds are needed to anticipate production yields and costs and prevent loss of productivity during industrial upscaling.

(ii) Technology is not a crucial limitation for industrial production of microalgae, but rather a rapidly evolving field that drives and stimulates innovation in microalgae markets. Increasingly efficient production techniques and processes have been developed, even on an industrial scale for demonstrators, but industrial profitability is often difficult to achieve, especially when there is already a competing resource established at lower cost [11,12]. For these reasons, research and development on microalgae is gradually moving towards the production of high-added value products based on biorefinery methods and investigates the development of cutting-edge applications and markets, rather than large-scale production of biomass for still highly competitive sectors such as energy (biodiesel). These innovative developments particularly concern the sectors of human and animal health, with applications including the production of recombinant proteins (vaccines, hormones), the purification of microalgae pigments for medical diagnosis, the production of innovative biomaterials and bioplastics, the use of microalgae extracts and metabolites for the prevention, diagnosis and treatment of cancer, neurodegenerative diseases and infections and the dermocosmetic sector with the development of photoprotective compounds, biocompatible pigments and bioactive ingredients [11,13,14,15,16,17,18,19,20,21,22,23,24,25,26,27,28,29]. 

(iii) The legislative framework is the most critical obstacle limiting the industrial development of microalgae. There is an important need to improve the knowledge of legislative staff on microalgae, to create ecological footprint certifications, to define regulatory frameworks and models for the importation and traceability of microalgae strains under the Nagoya Protocol and to define the concept of “domestic algae” for regulatory actions. With regard to food and feed legislation, it was suggested to produce a model document for the production/marketing of novel foods and to define the ingredients derived from GMO microalgae that could be added to the list of novel feeds. From an administrative point of view, it was noted that there was a need to develop and strengthen networks among microalgae stakeholders and to harmonize administrative procedures in the areas of agriculture, aquaculture and biotechnology.

(iv) According to experts, researchers, producers and stakeholders, the main promising markets expected for microalgae in the coming decades are the production of proteins for food and health applications, including vaccines and recombinant proteins, the production of polysaccharides and antibiotics and the use of microalgae for bioremediation and biofertilization.

## 2. Results

### 2.1. Delphi Analysis of the European Atlantic Area Microalgae Sector

#### 2.1.1. First Delphi Round: Electronic Survey of European Microalgae Stakeholders

##### Interviewed Stakeholders

**Figure 1:**
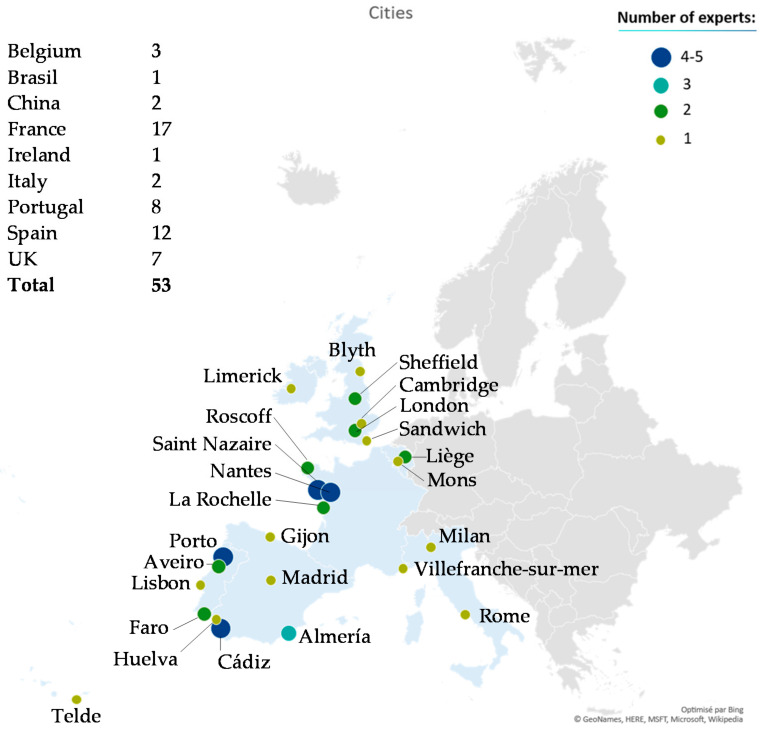
Map and repartition of survey respondents’ nationalities.

A good participation and representation of researchers and stakeholders were crucial to obtain relevant data to make a diagnosis and propose recommendations to boost the microalgae sector. A total of 53 experts responded to the online survey. As expected, most of these experts (82%) came from the Atlantic Area (AA) countries and mainly from France, Spain, Portugal and the United Kingdom, which made it possible to identify the main challenges and opportunities of microalgae in the European Atlantic territory. As the survey was disseminated on the Interreg EnhanceMicroalgae website, the opinions of international microalgae stakeholders outside the European AA were also collected, including colleagues from China, Brazil, Belgium and Italy. Due to the number of responses, these opinions were retained in the data processing but isolated from the general panel for AA-specific questions. On the basis of the mapping of the geographical distribution of the experts, sorted by country and city, a homogeneous distribution of experts over the territory was highlighted, extending along the Atlantic coast from Cadiz in the south to Blyth in the north (Figure 1). The mapping identified four main centers, namely, Saint Nazaire and Nantes in France, Porto in Portugal and Cadiz in Spain, with four to five responses collected in these cities. The rest of the opinions were spread over the territory with an average of one to two responses per institution.

**Figure 2:**
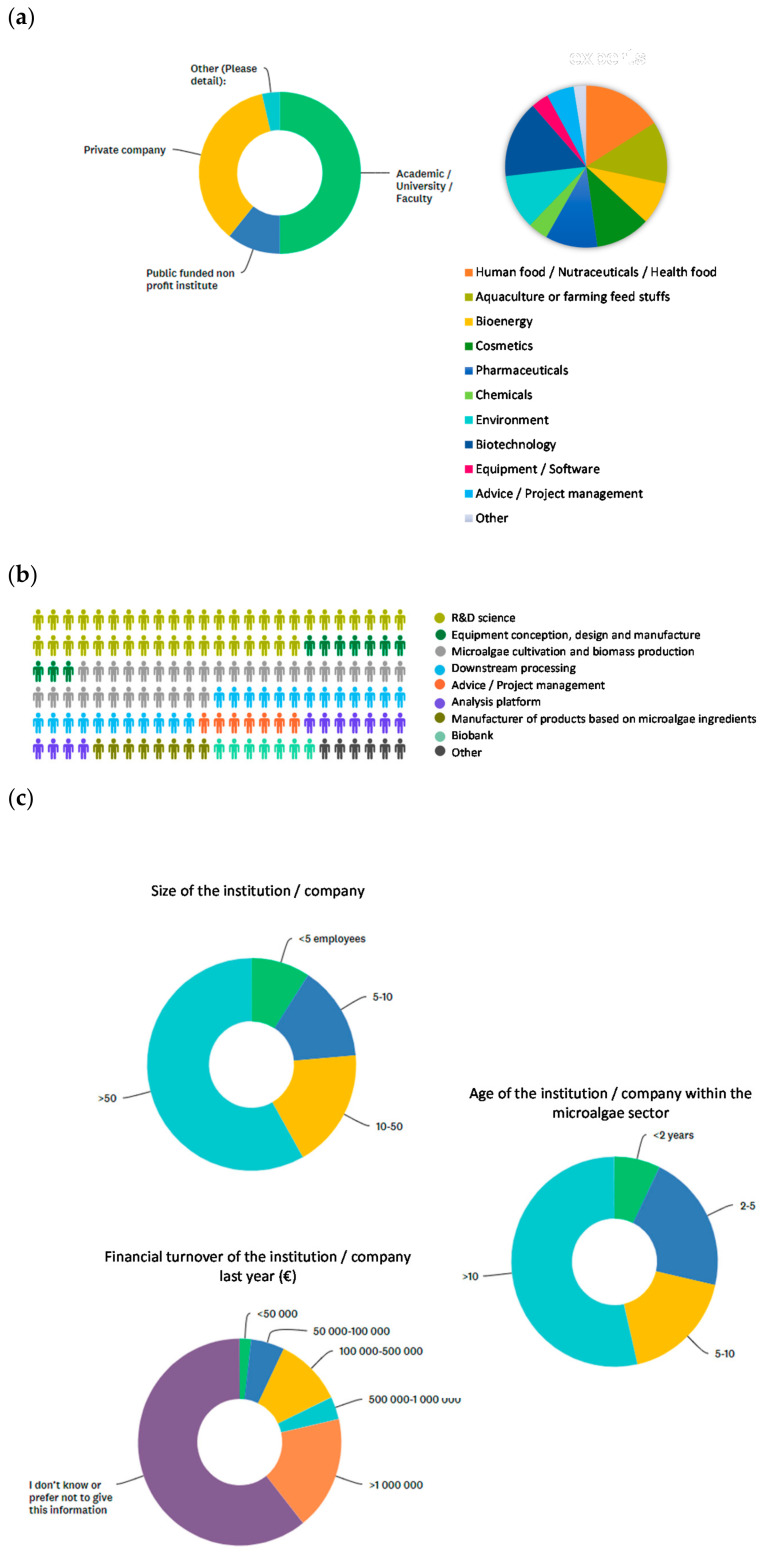
Sectors and domains of activity of surveyed stakeholders and characteristics of the institution/company of surveyed researchers and stakeholders. (**a**) Origin and domain of activity of surveyed stakeholders; (**b**) sector of activity and business of surveyed stakeholders; (**c**) institution/company of surveyed researchers and stakeholders.

Beyond their geographical distribution, the fields of activity and expertise of the panel of microalgae stakeholders interviewed were also varied. They belonged to both public and private organizations, with 51% of them working in the public sector such as an academy, university or faculty, 37% in a private company and 11% in a public non-profit institute (Figure 2a). All fields of activity were represented in the stakeholder panel, with 5–10% of them working in the food, nutraceuticals and health food industry, in aquaculture or animal feed breeding, in bioenergy, cosmetics, pharmaceuticals, environment and biotechnology and in consulting and project management. Less than 5% of them worked in the chemical industry, equipment/software or other sectors (Figure 2a). Respondents were mainly specialists in research and development, cultivation, production and downstream processing of microalgae biomass (Figure 2b). The high degree of multi-disciplinarity of the survey panel was crucial for the comprehensive collection of opinions. Finally, information was also collected on the size and age of each institution or company, indicating that they were mainly characterized by more than 50 employees and 10 years of longevity. The financial turnover of the institution was mostly not answered (Figure 2c). In conclusion, the respondents to the survey belonged to a varied scientific workforce with different skills, working in various institutions and with expertise in a wide range of sectors, which confirms the methodological quality of the survey's sampling.

##### Analysis of the Survey’s Answers

- Situation of the European AA in the global microalgae market

**Figure 3:**
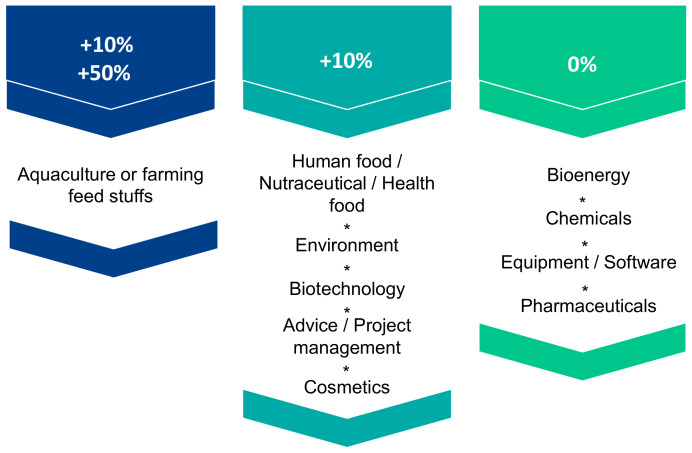
Contribution of the European AA to the global microalgae markets as perceived by the surveyed AA stakeholders.

**Figure 4:**
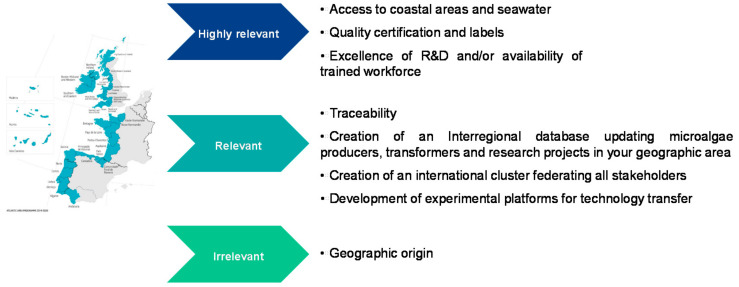
Advantages positively differentiating the AA from other European areas ranked by the surveyed stakeholders as highly relevant, relevant and irrelevant.

The European AA territory extends from the north of Scotland to the south of Andalusia and covers five countries including the Atlantic islands (Canary Islands, Madeira and the Azores). Stakeholders were questioned on the current positioning of this territory in the global microalgae market. As illustrated in Figure 3, the AA is perceived as contributing to approximately 10–50% of the world aquaculture and farmed animal feed market and 10% of the food, nutraceuticals, dietetic food, biotechnology, environment, cosmetics and consulting or project management markets. On the other hand, the contribution of this territory to the world markets of bioenergy, chemical industry, equipment, software and pharmaceuticals is estimated to be close to 0% according to the opinion of the stakeholders, which indicates a large possibility of progression. Stakeholders felt that the AA origin had benefits in positively differentiating microalgae products produced in its territory in the future. Access to clean coastal areas and seawater, product certification and quality labels, excellence in R&D and the availability of a trained workforce were among the most relevant criteria cited (Figure 4). Among the actions to be carried out to promote the AA and stimulate the industrialization of microalgae, the promotion of product traceability, the creation of an inter-regional database referencing and updating production/processing/research projects in the microalgae territory and a federation of all the actors of the AA within an international cluster were pointed out. The development of experimental technology transfer platforms was also highlighted as being crucial for the promotion of the sector. To this end, we recently built and published a database of AA microalgae actors (map and Excel database), accessible free of charge at https://www.enhancemicroalgae.eu/stakeholders-database/, in order to improve access to up-to-date information and to foster cooperation between companies and public institutions throughout the sector. According to the stakeholders interviewed, the geographical origin of the AA was not identified as an asset to differentiate microalgae products from other European producers. 

Both the AA and Europe should continue their scientific and technological progress by maintaining the level of training, R&D and increasing the establishment of larger-scale cultures such as experimental platforms. The main asset of the AA to create future opportunities was linked to the establishment of a federation of actors of this territory, through clusters and databases, and also to the implementation of traceability of microalgae products, ensuring their quality through certifications and labels.

- Overview of barriers limiting the development of microalgae research and markets in the AA

**Figure 5:**
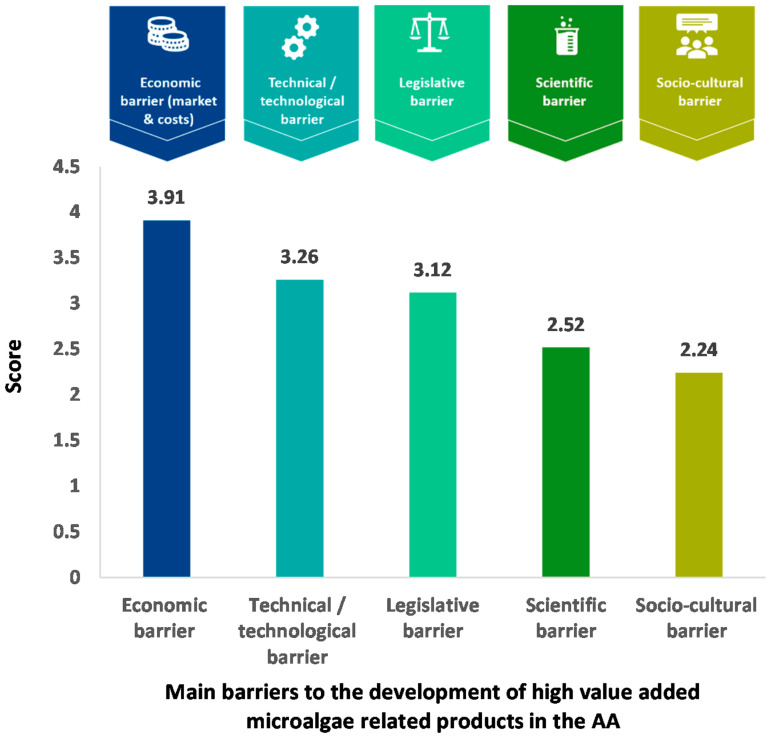
Main barriers to the development of microalgae and related products in the AA as perceived by the surveyed stakeholders.

The opinion of the stakeholders interviewed on the main obstacles limiting the industrial development of microalgae in the AA is presented in Figure 5. The economic issue was considered, by far, the most limiting according to 49% of the stakeholders, with a score of 3.91. This barrier is related to the market and production costs. In the second and third positions limiting the development of microalgae, 29% of the stakeholders ranked technical and legislative obstacles with a score close to 3.2. These barriers concern production equipment and capacity, purification technology and legislative regulation of microalgae, such as the use of microalgae in human food, in novel foods, in dietary foods or for environmental applications. Finally, other issues concerning scientific expertise in research and development and socio-cultural acceptance of microalgae were considered less important by stakeholders, with scores below 2.6 given by 36% and 45% of the experts, respectively. In conclusion, the survey highlighted that economic and technical progress may be the two most effective levers perceived by stakeholders to stimulate the microalgae sector in the European AA. On the other hand, science and consumer acceptability of microalgae are not seen as obstacles to the development of the sector and markets.

- Economic barriers

**Figure 6:**
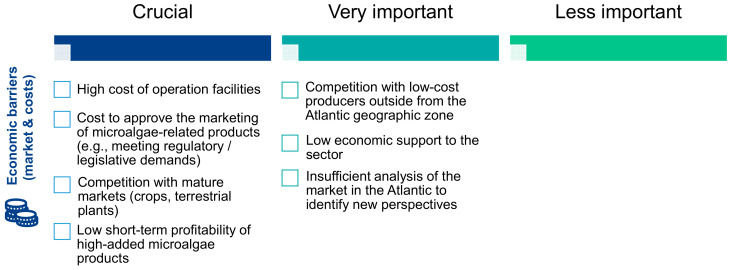
Importance of economic barriers according to the surveyed stakeholders.

The survey pointed out that the economic barrier was the greatest limitation to the development of microalgae in Europe and in the European AA. The high cost of operating facilities, the cost of approving the marketing of microalgae products meeting regulatory/legislative requirements, competition with mature markets (crops, land plants) and the low short-term profitability of products with a high microalgae content (Figure 6) were highlighted. Stakeholders also stressed the importance of competition with low-cost producers outside their geographical area, the low economic support to the sector and the insufficient analysis of the AA market to identify new opportunities. No economic barriers were identified as being of minor importance.

- Technical and technological barriers

**Figure 7:**
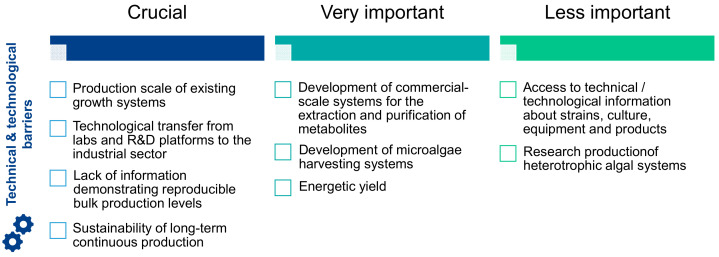
Importance of the technical and technological barriers according to the experts.

**Figure 8:**
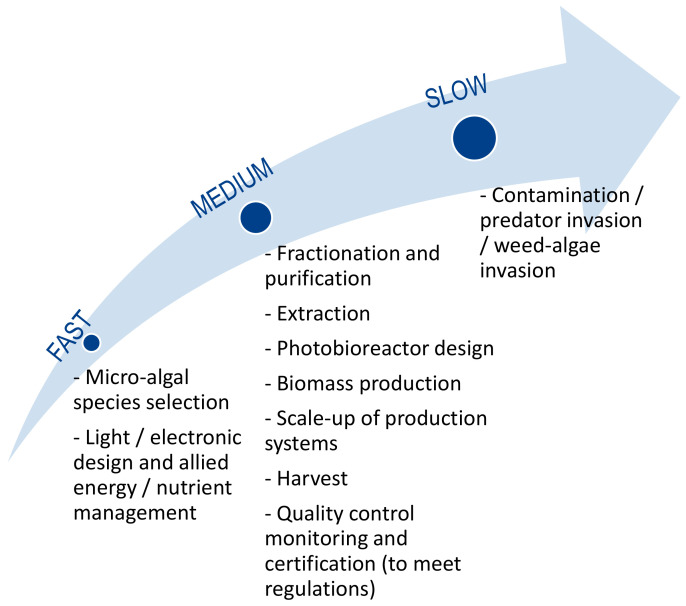
Evolution of the technical/technological barriers according to microalgae stakeholders.

The technological and technical gaps identified by stakeholders were ranked in order of importance (Figure 7). According to more than 56% of the respondents, the most important obstacles are the strengthening of existing growth and production systems for technology transfer from laboratories and R&D platforms to the industrial sector, the lack of information demonstrating reproducible levels of bulk production and the sustainability of continuous production in the long term. It was concluded that a specific effort should be made on these obstacles to stimulate the industrialization of microalgae. Other barriers classified as very important by stakeholders should also be considered such as the development of commercial-scale systems for the extraction and purification of metabolites, the development and innovation of microalgae harvesting systems and the improvement of energy efficiency in production processes. The following technical and technological issues were identified as less important by the experts: access to technical/technological information on strains, culture, equipment and products, and the development of heterotrophic algae growth systems. Stakeholders assessed the speed of change of the technological and technical barriers, and their opinion indicated that some were evolving rapidly and could be overcome fairly quickly, while others were evolving very slowly. Strain selection, light/electronic design and related energy/nutrient management were identified as fast-moving areas where limitations would be overcome quickly. Biomass production, harvesting, fractionation and purification, photobioreactor design, quality control, regulatory oversight and certification and scale-up of production systems were identified as medium-speed evolving areas. Finally, contamination/invasion of predators/invasion of harmful algae appeared as technological barriers that would take longer to overcome according to more than half of the participants questioned (Figure 8).

- Legislative barriers

**Figure 9:**
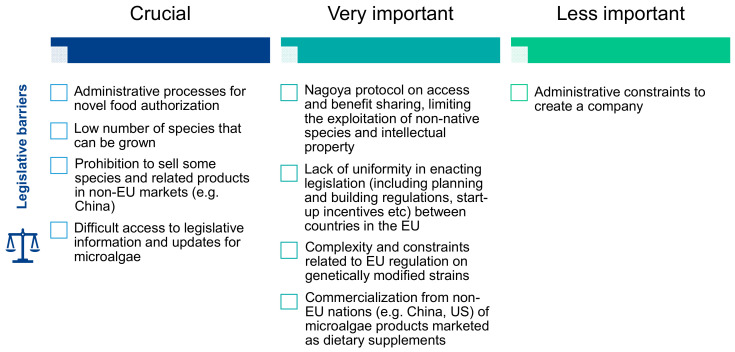
Importance of legislative barriers according to the interviewed stakeholders.

The legislation on microalgae was one of the three main obstacles identified as limiting the industrial expansion of microalgae. Stakeholders indicated that legislation on microalgae is complex, suffers from a critical lack of specialized personnel and requires special attention in the training of policymakers. Eighty-one percent of stakeholders interviewed on this issue highlighted the lack of specific legislative skills or training for scientists dealing with legislative and marketing issues in the microalgal sector. Legislation is evolving rapidly and is interconnected with other laws, which requires skills that go beyond those of scientists. The complexity of the authorization procedures for novel foods, the low number of species that can be cultivated, the prohibition on selling certain species (and related products) in markets outside the EU and the difficulties in accessing legislative information and updates on microalgae are among the crucial problems identified to develop markets for microalgae (Figure 9). It was pointed out that investors in the microalgae sectors could hardly obtain a clear picture of market regulations and that significant simplification of legislation could help the development of microalgae markets. Other legislative obstacles identified as very important by stakeholders concerned the Nagoya Protocol with the gaps in access and benefit sharing, limitations on the exploitation of non-indigenous species and intellectual property and the complexity and constraints of EU regulation on genetically modified strains. Laws relating to foreign countries are also seen as limiting, such as the lack of uniformity in the enactment of legislation between EU countries (including planning and building regulations, start-up incentives) and the marketing from non-EU nations (e.g., China, USA) of microalgae-related products marketed as food supplements. On the other hand, the administrative constraints associated with setting up a business were not identified as an obstacle to the development of microalgae markets.

- Scientific barriers

Scientific research was not identified as one of the main obstacles to the development of microalgae in the European AA by the stakeholders interviewed. The perception is that the European AA (and more broadly Europe as a whole) benefits from the scientific excellence of research and industry in the microalgae sector and plays a key role in the conduct of microalgae research and development. However, scientific research aimed at reducing production costs must continue, particularly with a view to inventing more efficient cultivation processes, optimizing photobioreactors and improving species to increase production yields of high-added value molecules. Future scientific challenges will be to improve the genetic data of microalgae, to prevent and reduce the risks of contamination by improving knowledge of contaminants and to better analyze and understand the composition of algal biomass. Among the main current scientific obstacles, stakeholders also highlighted the lack of scientific demonstration of the biological activity of microalgae-related products, insufficient supporting investments in industrial and academic consortia and insufficient public funding for R&D.

- Socio-cultural barriers

European consumer interest in microalgae, cyanobacteria and related products has increased very significantly in recent decades, in parallel with the consumption of related food supplements (in particular, the biomasses of *Spirulina/Arthrospira*, *Chlorella* and related products). AA stakeholders consider that there are no socio-cultural barriers limiting the development of microalgae products and applications in Europe, with the exception of genetically modified microalgae. However, minor socio-cultural barriers could still limit market development, including the lack of information and knowledge of potential consumers, the validity and reliability of information sources for consumers, consumer fears that microalgae may be contaminated by pollutants, heavy metals and/or toxins and the lack of visibility and communication of microalgae businesses with consumers (e.g., in social networks). With regard to the low consumer acceptability of GM microalgae and related products, rational identification of market sectors that could develop these products is likely to overcome consumer reluctance. For example, while acceptability is likely to remain zero for human or animal food applications, the use of marine microalgae for the production of therapeutic recombinant proteins could be quickly accepted with rational explanations.

#### 2.1.2. Second Delphi Round: Workshop with European Atlantic Area Microalgae Experts

The conclusions of the survey were presented to a panel of thirty scientists and industrialists from Spain, Portugal, France and the United Kingdom, considered to be European experts on microalgae and cyanobacteria. The objective of the discussion was to validate the perception of AA actors by experts who have an integrative vision of the sector and who are involved in the management of European networks of microalgae biomass producers, important research platforms and production/processing companies. A discussion was opened on each gap and obstacle limiting the development of the microalgae sector in the European Atlantic Area and the experts were invited to propose guidelines and recommendations to overcome these limitations.

##### The Most Critical Barrier according to Experts: Legislation

**Figure 10:**
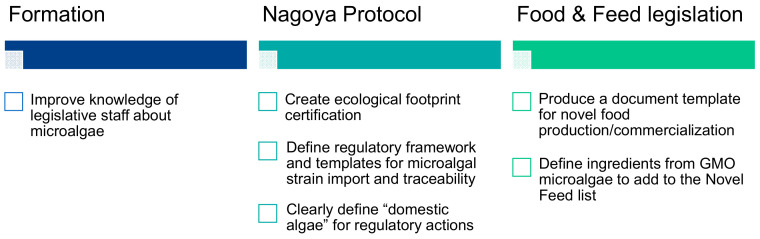
Main legislative gaps identified by the European microalgae experts and related proposed guidelines to overcome legislative gaps.

There was a consensus among the experts that legislative issues are the most critical obstacles to the industrial development of microalgae. These legislative obstacles have been analyzed in depth by the partners of the Interreg EnhanceMicroalgae program in charge of Workpackage 6 (University of Porto, CIIMAR), and their detailed conclusions can be read in a recently published paper [10]. The main limitations related to legislation identified by the experts include (i) the small number of species that can be cultivated compared to the immense biodiversity of species and strains, (ii) the complexity of administrative processes for the authorization of novel foods, (iii) the difficult access to information and legislative updates for research and commercialization of microalgae, (iv) the ban on the sale of certain species and related products in non-European markets (e.g., China) even though they are allowed in Europe and (v) the lack of uniformity in the enactment of legislation between EU countries, including planning and building regulations and start-up incentives. As it is shown in Figure 10, these legislative limitations could be divided into three packages corresponding to the lack of legislative training of specialized staff, limitations related to the Nagoya Protocol and improvements in food and feed legislation. 

According to the experts, legislation on microalgae suffers from a lack of specialized staff and requires special attention in the training of policymakers. European markets for microalgae could grow rapidly if legislative changes were made to fill the gaps in the legislation. However, the experts stressed that legislation cannot be drafted by scientists because of its complexity and the rapid and interdependent evolution of laws. The experts therefore recommended that the knowledge of legislative staff on the applications of microalgae should be improved by stimulating exchanges between scientists and lawyers. The Nagoya Protocol on Access to Genetic Resources and Fair and Equitable Sharing of Benefits Arising from their Utilization (below designated as “Nagoya Protocol”) is an international agreement that aims at sharing the benefits arising from the utilization of genetic resources in a fair and equitable way. Implemented in 2014, it raises a lot of questions about the industrial and academic development of microalgae in Europe, particularly about the accurate definition of species and strain origins, possibility to use species and strains isolated from foreign environments and industrial protection of these strains. The major obstacle in relation to the Nagoya Protocol concerns the accurate definition of “domestic” organisms. The question is to define how long is needed for an algal strain or species grown in a photobioreactor to be considered as domestic or acclimated, and this issue would need to be clearly answered for regulatory actions. A major challenge to the industrial development of microalgae in Europe is also to face competitive production such as the Asian one. In particular, as highlighted by two experts from the European Algae Biomass Association (EABA), the legislation on organic products is unclear since the organic certification does not provide information on the origin of the country producing the algal biomass and it is easy to produce a false certificate. The only solution proposed by experts would be the creation of a product certification including an ecological footprint. Additionally, Europe should define a framework and template for microalgae strain import, traceability and quality control. Currently, the legislation to prevent the free movement of strains and biomass between countries is only limited by the Nagoya Protocol and by material transfer agreements (MTA). As suggested by the European Algae Biomass Association (EABA), promoting the creation of a quality standard by the EU Standard Association Comity would help control the taxonomic, microbiological, toxicological and biochemical quality of strains and biomass and their traceability. Networks of collaborations are trying to solve this problem, for example, in the UK, the Phyconet network is working on the risks associated with the introduction of new strains in the UK by creating a new legislative framework and training politicians on microalgae. The aim is not only to achieve differentiation between EU producers of algae and importation from other countries related to food safety but also to create a tool in the EU to differentiate within an algal biomass produced exclusively in Europe and an algal biomass produced or imported by a European stakeholder in an external EU country with low production cost. In particular, some European joint venture companies already produce or buy microalgae in Asia that finally feed the European market and are sold as “Made in Europe” products. Biomass produced out of Europe may not reach the EU quality standards in terms of purity and toxin content, and microalgae-related products might contain a mix of synthetic and natural molecules (e.g., carotenoids), making traceability difficult as few laboratories have the analytical capacity to differentiate them. 

Regarding the food and feed legislation, the experts proposed that Europe should define and produce a document template to help companies obtain authorization for the production and commercialization of novel foods. Currently, a specific application submitted to EFSA is required for each product, and the analytical tests cost between EUR 200,000 and 300,000 for each product (these analyses are very extensive, require an average delay of 2 years and must be performed in certified toxicology laboratories). An improvement would be that the European Commission identify 20–30 species and state the conditions that must be used to grow them to reach the toxicological quality required by the EU. Starting from this biomass, extracts could be authorized for novel food production and commercialization. By creating a standard operating procedure, companies wishing to introduce new microalgae and related products in the market would also be informed about the mandatory toxicological, genetic and physiological analyses and could select species among a list of 20 to 30 already authorized species. 

The second point concerning food and feed legislation concerns the use of genetically modified microalgae as a possible source of feed ingredients. Currently, feed formulators can include microalgae listed in the feed catalogue as ingredients. Genetically modified microalgae are prohibited, but there is no certification or testing to prove that GM microalgae are actually absent from the final product. EABA, in agreement with the European Commission of the Regulatory Council, considers that genetically modified microalgae can be defined as strains and species that do not exist in nature. These strains are generally obtained by insertion/modification of genes using plasmids and genetic recombination, such as the CRISPR-Cas9 technology. The question is whether these genetically modified strains can be accepted for animal feed, added as an additive or ingredient at a maximum final concentration in the product, and whether improved strains obtained by a mutagenesis-selection process (induced by UV treatment or otherwise), although being obtained by non-targeted mutation, should be classified as genetically modified microalgae or not. 

The experts underlined the lack of consistency and precision regarding national authorizations for cultivation and research related to synthetic biology, not only for microalgae but also for vectors and plasmids. The European Court of Justice decided in July 2018 to classify gene insertion, gene editing, CRISPR-Cas9 and relative recombination technologies as leading to GMOs, but, for example, in France, the mutagenesis-selection process is not included in this category as no targeted genome modification is produced by this selection strategy.

##### Scientific Gaps

Regarding scientific gaps, the experts confirmed the lack of genetic data on microalgae species. Microalgae represent a largely untapped reservoir of new and valuable species for the production of bioactive compounds and the elucidation of their biosynthesis pathways is an expanding field of research, in order to reduce production costs and increase yields for the production of high-value-added molecules. Initially focused on biodiesel production studies, metabolic research is now focusing more on the biosynthesis of other biomolecules such as antioxidant, antiviral, antibacterial, antifungal, anti-inflammatory, anti-tumor and anti-malarial compounds. The advent of cost-effective next-generation sequencing platforms (NGS) now makes it possible to produce a large amount of genetic information, and the sequencing and annotation of genomes makes it possible to elucidate the pathways of biosynthesis and genome transformation. However, several limitations persist in terms of genetic knowledge. At present, companies that transform microalgae genomes are facing problems because many microalgae genomes are not yet sequenced, forcing them to sequence the genome on their own. Another major problem linked to the rapid expansion of sequencing technology is the presence of automatic annotation errors and the lack of European scientific experts to work on and correct annotation errors. As a result, the amount of genetic data is very large, but there is a lack of information on the annotation of the genome, and scientists are very likely to work with incorrect data, leading to a series of errors.

The second major scientific problem for Europe and the AA is the contamination of microalgae cultures in large-scale production by bacteria, viruses and fungi. The productivities of the cultures must remain stable from laboratory to industrial scales, but generally, contamination problems occur during the scaling-up process and when switching to open systems. Contaminants are not well studied and identified, and a scientific effort must be made to improve knowledge and data in omics concerning the interactions of microalgae with microorganisms (bacteria, fungi or others) that modify productivity. The problem of large-scale contamination affects all countries but may not be examined in detail by low-cost producers who can compensate for a loss of productivity and quality with high volumes. Instead, the approach of Europe and AA experts is to target high-quality production combined with scientific knowledge to improve crop biocontrol, understand inter- and intra-species interactions and prevent contamination. 

Scientific demonstration of the biological activity of microalgae extracts and purified products is another future scientific challenge for Europe. To ensure rapid development and profitability of their products, companies need reliable tests for rapid proof of concept and robust demonstration of the biological activity and efficacy of their metabolites of interest. This step requires a wide range of scientific expertise and equipment, as different activities are evaluated and it is expensive research, sometimes difficult for a small SME to carry out. Proof of concept is a key point necessary to develop, in particular, the use of microalgae-related products in the pharmaceutical and nutraceutical sectors. With this objective, the development of public research platforms represents a driving force that will benefit private companies, by developing new tests, screening platforms, standardization and model validation to prove the biological activity of microalgae-related products. 

**Figure 11:**
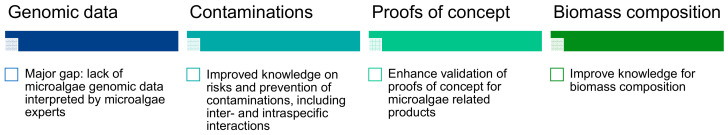
Main scientific gaps identified by the European microalgae experts and proposed guidelines to overcome these gaps.

The final scientific hurdle reported by experts is the lack of knowledge on the biomass composition of microalgae, and the need to improve the analytical characterization of species to target new bioactive molecules. A summary of the scientific gaps and obstacles and the experts' recommendations is presented in Figure 11.

##### Marketing Gaps

**Figure 12:**
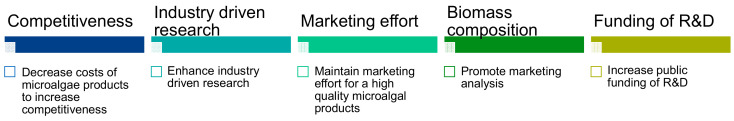
Main marketing and commercial gaps identified by the European microalgae experts and proposed guidelines to overcome these gaps.

The experts confirmed that the microalgae market in Europe suffers from commercial and marketing shortcomings (Figure 12). Europe has to compete with low-cost international production and it is clear that the low cost of Asian production cannot be achieved in Europe. The introduction of taxes for the import of microalgae, improving the profitability of European production by lowering production costs or stimulating research by industry can help to reduce the trade gap between European and Asian production. It was also pointed out that European instruments and funding for research are not always adapted to the needs of industry. The time needed to prepare and submit a proposal does not correspond to that of industry, as the market changes very quickly while waiting for European research funding. This issue should be examined and discussed at the European level for future programs. At the national level, programs are faster (and smaller) and, in many cases, more adapted to the needs of a specific industry. Therefore, although this is a general problem not specific to algae but also to other sectors, it affects research performance and the transfer of research results to industry and markets. The microalgae sector in Europe needs knowledge transfer tools, such as acceleration programs to support the creation of spin-offs and start-ups, to promote skilled employment in the microalgae sector and to ensure the sustainability of transfer programs. A marketing effort is also needed to maintain the quality of microalgae products, which is clearly the differentiating factor of European production, as well as its social and environmental quality. Europe should encourage consumers and businesses to focus on the quality and origin of microalgae-related products and to mark products in this way by clearly indicating the origin of the product with labels to stimulate local consumption. Another point concerning marketing is the need to promote marketing analysis with a solid business plan including public perception of products, identification of the best products for a company and the suitability of products and production facilities to reach the market. An important issue that should also be considered is how to stimulate the promotion of new and innovative products with high marketing potential. Finally, the experts highlighted the difficulty for companies to obtain financing, especially for small companies that want to move from laboratory to industrial scales, and the need to increase public funding for R&D.

##### Administrative Limitation, Research Network and Social Network Impacts

**Figure 13:**
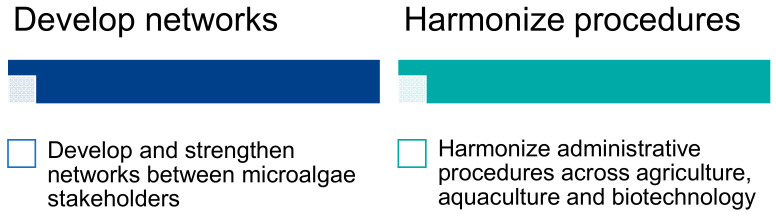
Main administrative gaps and guidelines identified by the experts during the workshop in La Rochelle.

The experts mentioned the importance of communication between researchers and industrialists to foster the European microalgae sector. Regional and European networks already exist in the AA (e.g., EABA, Phyconet, COST) and should be supported to strengthen collaboration networks between actors in the microalgae sector and to harmonize administrative procedures (Figure 13). Some experts stressed that the number of such networks should remain low to avoid dilution of funding and information, and to centralize connections between academia and industry. The use of social platforms and tools is an influential social key for the development of microalgae (LinkedIn, Twitter, Facebook, website, etc.). EnhanceMicroalgae partners mentioned the creation of brokerage activities and an online marketplace (http://emamarket.anfaco.es) dedicated to AA actors in the field of microalgae in order to facilitate and strengthen the network of collaborations. In addition, they also created a stakeholder database for the European Atlantic Area which can be very useful to find a research or industrial partner with specific skills and equipment (https://www.enhancemicroalgae.eu/stakeholders-database/). Currently, applications of microalgae-based products cover the agriculture, aquaculture and biotechnology markets, and the experts recommended that a European harmonization of administrative procedures for these sectors could be useful for microalgae-related products.

##### Technological Gaps

**Figure 14:**
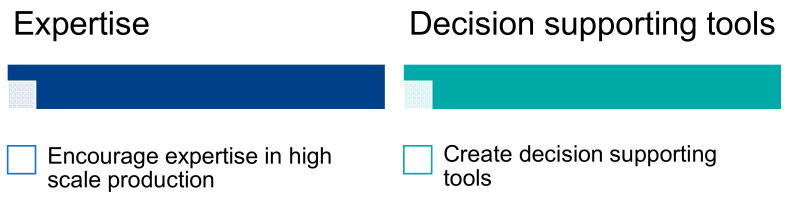
Main technological gaps identified by the experts during the workshop in La Rochelle and related guidelines.

Experts said that Europe has some of the best companies, institutes and start-ups innovating in technology development, including processes and equipment for growing, harvesting and processing. Therefore, technology was not identified as a significant barrier to the development of microalgae research and markets in Europe and the AA, but rather as a crucial differentiating advantage for AA laboratories and companies. However, most people working on microalgae operate at the laboratory scale, and the lack of expertise in large-scale cultures and facilities probably contributes to limiting the industrial development of microalgae. It seems important in the future to train staff at all stages of industrial production and to encourage expertise in large-scale production (Figure 14). Europe should also support the acquisition of large-scale equipment to improve training in the cultivation, harvesting and processing of microalgae. Some companies do not have the facilities and expertise to develop innovative processes such as supercritical extraction or membrane technology, for example. These companies therefore need academic support, and to this end, the creation and dissemination of accessible databases on microalgae facilities, actors and contacts in Europe and in the AA will help them overcome the technological barriers. In addition, the creation of decision support tools (DST) based on in silico simulation platforms for microalgae growth and metabolite production will also help them to plan and optimize commercial biomass production according to their needs and commercial objectives. An example of such a DST has been developed by Partner 7 of the Interreg EnhanceMicroalgae program (Swansea University) and can be found at https://www.enhancemicroalgae.eu/decision-support-tool/ [30].

##### Promising European Microalgae Markets

**Figure 15:**
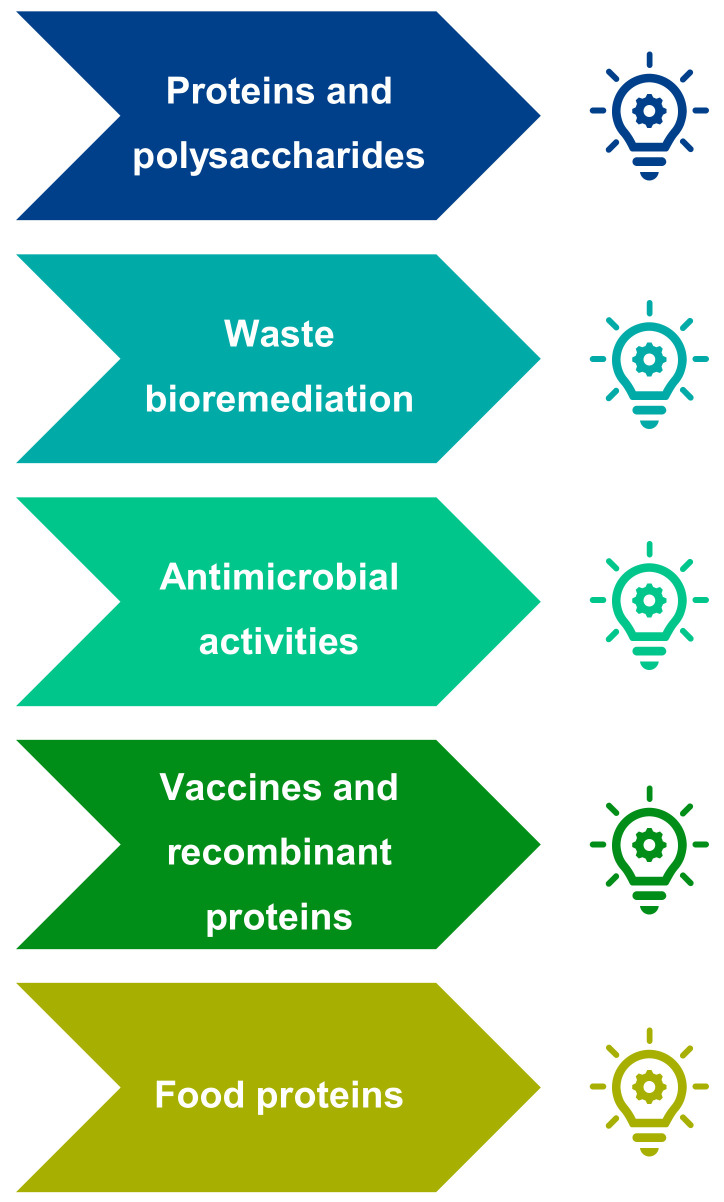
Identification of five microalgae sectors that could meet significant market growth in the near future, according to AA microalgae experts.

The world is facing challenges related to food supply and safety, energy, health and environmental issues. Research on microalgae could offer solutions to many of these challenges, leading to the development of promising markets. The experts identified four sectors that they believe will meet significant growth in the near future: protein and polysaccharide production (with a particular focus on food proteins for food sustainability), bioremediation of waste and the use of microalgae as biofertilizers, the development of new antibiotics and antimicrobial compounds and the production of vaccines and recombinant proteins (Figure 15).

- Proteins and Polysaccharides

Among the most promising markets in Europe, the production of microalgae proteins and polysaccharides was highlighted by the experts. There is, indeed, a growing demand for these molecules to meet food sustainability, to replace animal proteins by vegetable proteins and to meet the food processing needs of the food industry. Unlike the lipids and pigments sector, where the promising markets for microalgae will be rapidly overtaken by synthetic molecules or natural molecules produced by high-yield producers such as the marine fungus *Schizochytrium*, the simplest and most cost-effective way to produce microalgae proteins and polysaccharides is still to grow the biomass and fractionate it. Microalgae proteins and polysaccharides are of high nutritional, organoleptic and functional quality and can be produced together with PUFAs, vitamins and antioxidants to develop food supplements and extracts. The global demand for primary metabolites of microalgae (proteins, polysaccharides, lipids) will increase fivefold in the coming decades, to feed the world and meet the demand for replacing animal meat with plant products. From the point of view of industrialists and consumers, microalgae also offer significant advantages over other biomasses: high growth yield per hectare, high metabolite production yield, no use of freshwater resources, continuous production with no fixed harvest period, no use of arable land and reduced use of phytosanitary products compared to land agronomy. The production of microalgae could also be a sustainable alternative to achieve food self-sufficiency for countries lacking arable land.

- Waste bioremediation and valorization of microalgae as biofertilizers

Microalgae can be used as an effective depolluting biomass for the bioremediation of water, soil and air. European companies in the AA are studying the treatment of urban or industrial wastewater and gases by microalgae on an industrial scale to remove nitrates, soluble and volatile organic pollutants or heavy metals. The proof of concept of a possible and realistic use of microalgae for large-scale bioremediation stimulates investment in this field, and it is possible to valorize polluted biomass for the production of biogas, ethanol and biohydrogen, particularly by using anaerobic digestion. Furthermore, microalgae have been identified as a relevant substitute for chemical fertilizers to increase soil fertility, and their use as biofertilizers will grow strongly in the coming decades, not only as a source of nutrients but also for the treatment of bacterial and fungal phytopathogens and to improve the nutritional quality of crops. Although the short shelf life of biomass and the need for cold storage may limit the development of microalgae as biofertilizers, experts estimated that the market would grow considerably in the coming years, in parallel with the ecological transition that should be gradually implemented in agronomy.

- Antimicrobial activities of microalgae and related products

Bacterial resistance to antibiotics is a major threat to human and animal health and alternative treatments to cure infections caused by resistant strains are urgently needed. Experts considered that microalgae could offer great potential to replace some of the antibiotics used in animal husbandry and aquaculture, and to identify innovative antibiotics for animal and human use, as well as innovative antibacterial compounds, such as photosensitizers for photo-decontamination and antibacterial phototherapy. Although companies are not investing sufficiently in the development of new antibiotics, the need and market for these molecules will increase dramatically in the coming years.

- Vaccines and recombinant proteins from GM microalgae

A final area in which microalgae will have a major role to play in the coming years is the production of vaccines and recombinant proteins. Experts are convinced that microalgae offer several advantages for use as gene expression systems for the production of cytokines, antibodies, hormones and vaccine peptides and proteins. The proof of concept that microalgae can be genetically transformed for the production of therapeutic proteins is now well established, but there is still a need to scientifically optimize the processes to ensure high and stable expression levels and adequate glycosylation. Faced with the emergence of major health crises linked to new viruses and pathogens, microalgae could prove to be the biotechnological tools of choice for the rapid and safe production of recombinant proteins, with improvements over existing production systems in terms of purification and prevention of contamination by prions or animal viruses.

#### Conclusions of the Workshop

The seminar in La Rochelle brought together international experts from industry and government to identify promising markets and discuss gaps to improve production, technology and marketing in the AA microalgae sector. The experts' reflections identified the main obstacles to the industrial development of microalgae in Europe and the AA and provided guidelines for an improved microalgae sector. 

**Figure 16:**
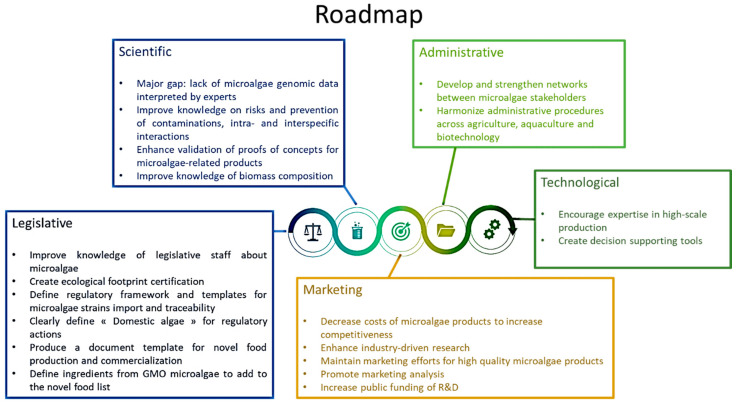
Roadmap to overcome gaps and barriers to the development of the microalgae sector in the European Atlantic Area, according to experts’ recommendations.

**Figure 17:**
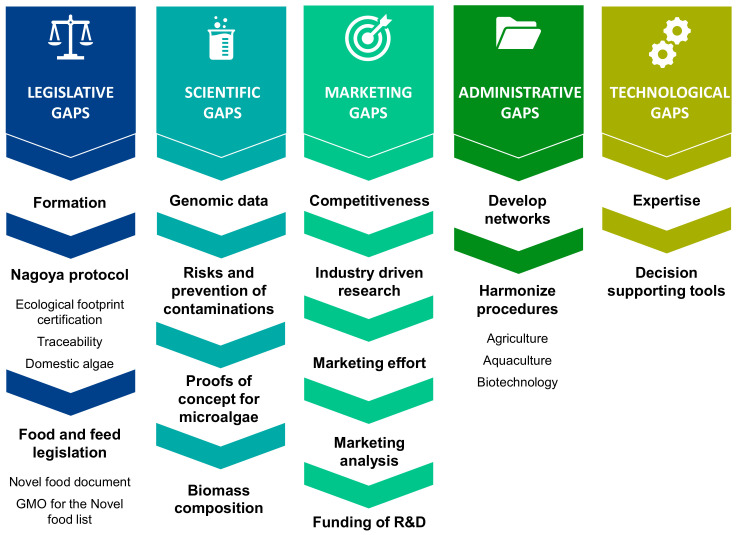
Microalgae experts’ guidelines for an improvement of the European microalgae sector.

All experts agreed that legislation was the most important obstacle to the industrial development of microalgae in the AA. The experts advised on the training of a qualified workforce, improvement of the Nagoya Protocol and improvement of food and feed legislation. They also pointed out that the lack and accuracy of genomic data, the problem of large-scale crop contamination, the need for proof of concept and the lack of knowledge on biomass composition were the main scientific obstacles. The experts also advised on measures to increase the competitiveness of microalgae markets through industry-led research, marketing efforts, market analysis and increased support for R&D. It was considered that administrative barriers could be avoided by developing more collaborative networks between actors in the microalgae sector and by harmonizing procedures between different application areas (agriculture, aquaculture, biotechnology). Finally, the technological gaps related to the production of microalgae require the training of a qualified workforce for industrial production as well as decision support tools. This report has provided a roadmap for the development of the microalgae sector (Figure 16), identifying strategies and guidelines for industries and public authorities to overcome technological, legislative and market barriers in the microalgae sector (Figure 17). The seminar also highlighted the opportunities to improve production, technologies and marketing in the AA microalgae sector by focusing on the market for microalgae-specific proteins and polysaccharides, the use of microalgae in bioremediation of waste and their valorization as biofertilizers and their potential use for antimicrobial/antifungal activities and for the production of therapeutic recombinant proteins.

### 2.2. SWOT Analysis

Based on the perceptions of European microalgae experts and European stakeholders, an updated SWOT analysis of research, development and commercialization of microalgae in the European Atlantic Area in 2020 was carried out (Table 1). Additional minor elements perceived by the authors of this publication were added to this SWOT analysis.

### 2.3. Traffic Lights Representation for a Fast Evaluation of Gaps, Risks, Barriers and Opportunities

Supplementary Data Table S1 presents a detailed traffic light analysis of the main scientific, technical, legislative, development cost and market gaps and barriers, as well as an overall assessment of near-future market opportunities for all microalgae sectors.

## 3. Discussion and Conclusions

European research and development on microalgae has significant space for improvement, and important market growth is expected in the coming decades. To target these market opportunities, current scientific, technical/technological, commercial and legislative gaps and barriers need to be accurately and comprehensively identified in order to put in place actions and guidelines to overcome these limitations. In this paper, we have carried out an in-depth analysis of the perceptions and recommendations of stakeholders and experts in the European Atlantic Area, in order to stimulate the development of the food, feed, health, cosmetics, nutraceuticals, biomaterials, pharmaceuticals and biofuels sectors. Among the main scientific gaps, the lack of genomic data on microalgae reliably interpreted by experts, the need to improve knowledge on the risks and prevention of culture contamination, including inter- and intra-species interactions, and the need to validate proofs of concept for microalgal products and to improve knowledge of the biomass composition of microalgae were highlighted. Technology was not identified as a crucial limitation for industrial production of microalgae, but rather as a rapidly evolving field that is crucial to stimulate innovation in microalgal markets. A need for expertise in large-scale production and access to decision support tools for the production of microalgae and related compounds was identified. The legislative perspective was identified as the most critical obstacle limiting the industrial development of microalgae, highlighting the need to improve the knowledge of legislative staff on microalgae, the need to create ecological footprint certifications, the need to define regulatory frameworks and models for the importation and traceability of microalgal strains under the Nagoya Protocol and the need to define the concept of “domestic algae” for regulatory actions. In addition, the low number of authorized species was highlighted, relative to the high taxonomic diversity of microalgae and cyanobacteria. With regard to food and feed legislation, it was suggested to produce a model document for the production/marketing of novel foods and to define the ingredients derived from GMO microalgae that could be added to the list of novel feeds. From an administrative point of view, it was noted that there was a need to develop and strengthen networks among microalgae stakeholders and to harmonize administrative procedures in the areas of agriculture, aquaculture and biotechnology. 

By integrating all the considerations of experts and stakeholders, it was possible to conclude that the main promising markets for microalgae in the coming decades are the production of proteins for food and health applications, including vaccines and recombinant proteins, the production of polysaccharides and antibiotics and the use of microalgae for bioremediation and biofertilization. Although many sectors of activity are still in their infancy, proper adaptation of legislative barriers could unlock the research potential of the European Atlantic Area, opening the door to new cutting-edge applications and markets that are not limited by science or technology.

## 4. Materials and Methods

### 4.1. Two-Round Delphi Analysis of Microalgae Researchers’, Experts’ and Stakeholders’ Opinions

#### 4.1.1. Definition of the Study Area: The European Atlantic Area (AA)

**Figure 18:**
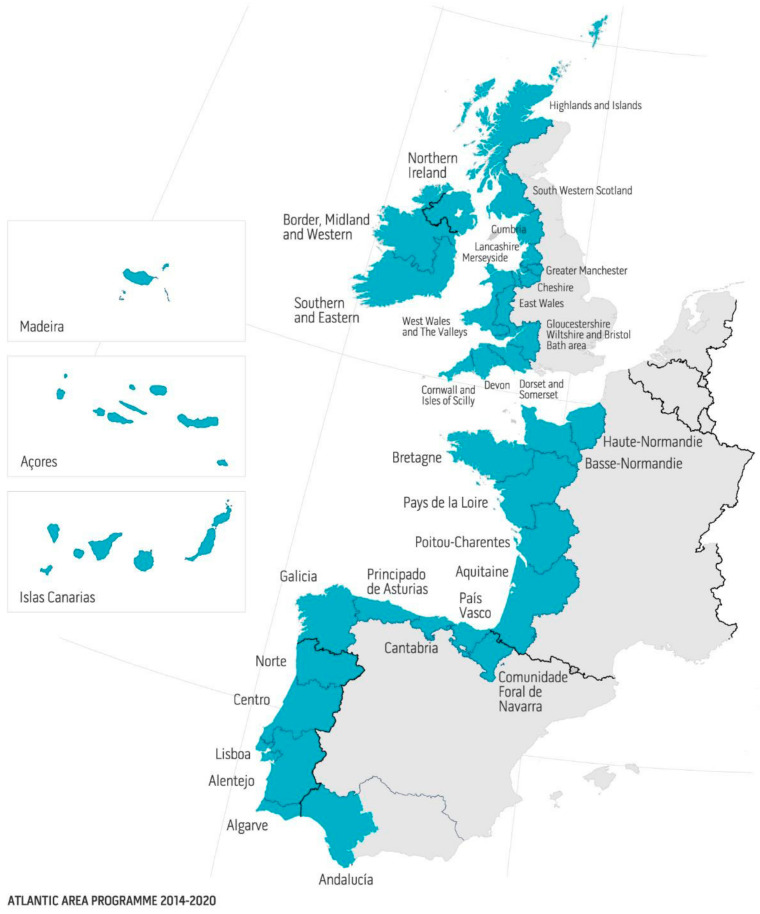
Map of the European Atlantic Area regions. The region included 36 coastline administrative regions from 5 countries (Portugal, Spain, France, UK, Ireland) benefiting from Interreg research funds.

This study was geographically limited to the European Atlantic Area that included 36 coastline administrative regions from five countries (Portugal, Spain, France, UK, Ireland) at the time of this research, as defined by the Interreg Atlantic Area funding program for the 2016–2020 period (Figure 18) [31]. The UK regions were included in this study because they belonged to this area and benefited from Interreg funds before the effective Brexit.

#### 4.1.2. First Round: Electronic Survey

**Figure 19:**
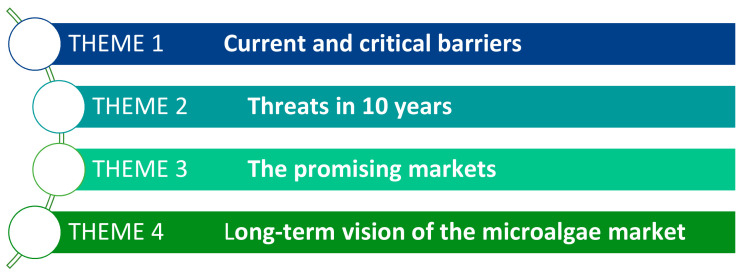
List of the 4 themes covered in the online survey to identify gaps and barriers limiting the industrial development of microalgae in the European Atlantic Area.

An online electronic survey was drafted to question stakeholders in the European AA, including researchers, experts and industrialists, on the sectoral challenges and obstacles to the industrial development of microalgae in the European AA. It included multiple choice questions, rating scales and open comments on existing and desirable developments in infrastructure, technologies, markets and legislation (see Supplementary Data Figure S1 for details of the survey questions). The data collection revealed convergence of opinions, consensus and areas of uncertainty. Designed with SurveyMonkey software, the survey asked stakeholders about the current limits of industrial development of microalgae in the AA, the main threats and opportunities for microalgal markets and their views on a longer-term vision for the microalgal market. The results of the survey were divided into and analyzed as 4 themes (Figure 19). The introductory questions aimed to describe the skills and sectors of activity of the actors interviewed as well as their nationality and type of organization. After situating the position of the Atlantic Area in the world market and highlighting its main assets, the first part focused on the current limits to the development of research, development and markets for microalgae, including the crucial obstacles in the economic, technological, scientific, legislative and social fields. The second part enabled stakeholders to highlight the threats to the development of microalgae in the Atlantic Area and Europe over the next 10 years. The third part was devoted to the identification of the next scientific breakthroughs that could allow the emergence of promising markets and the maturation of microalgae sectors. Finally, in the fourth part, questions aimed at providing a long-term vision of the microalgae market, and 5 key aspects for boosting the microalgae sector were highlighted, summarizing the main orientations resulting from the opinions of the stakeholders in this survey. For ranking questions, the average ranking of each answer was calculated as follows:

*w* = weighting of the assigned position;
*x* = number of responses for that answer choice;
Score = (*x*1*w*1 + *x*2*w*2 + *x*3*w*3+ … *xnwn*)/Total number of responses.

The weights were applied in reverse. This means that the response choice preferred by the participant (the one he/she ranks first) had the highest weighting (for example, 5 in the case of 5 possible answers), while the choice he/she least preferred (the one he/she ranks last) had a weighting of 1.

#### 4.1.3. Second Round: Workshop with Microalgae Experts from the European Atlantic Area

Following this survey, an international workshop dedicated to research, innovation and industrial development of the microalgae sector in the European Atlantic Area was organized in La Rochelle, France. These two days of meetings and exchanges took place within the framework of the European Atlantic Area inter-regional research program (Interreg EnhanceMicroalgae EAPA_338/2016). Thirty scientists and industrialists from Spain, Portugal, France and the United Kingdom, considered European experts in the field of microalgae and cyanobacteria, participated in the workshop. The invited personalities were selected on the basis of their expertise and impact in the field (assessed by their number of publications and their responsibilities in the management of European companies, networks and associations of microalgae producers), with the aim of achieving a balanced representation of the different countries, sectors of activity (research, development, industrial transfer, production, processing, marketing) and expertise (innovation, legislation, technology, fundamental research, etc.). The discussions were based on the results of the survey and aimed at drawing up an inventory of the sector, identifying gaps and obstacles limiting the development of the sector and drawing conclusions and recommendations/guidelines to facilitate research development, technology transfer, industrial development and transfer to markets.

### 4.2. SWOT Analysis of the Microalgae Sector in the European Atlantic Area

On the basis of comments and opinions provided by European experts and stakeholders, an updated SWOT analysis of the sector was carried out to highlight its strengths, weaknesses, opportunities and threats in 2020.

### 4.3. Traffic Light Analysis of Market Opportunities

Data provided by experts, researchers and stakeholders were integrated and combined with our previous data on scientific research that stimulates markets for microalgae and related products. For each sector of activity, a favorable (green light), moderately favorable (amber light) or unfavorable (red light) rating was given based on the identification of scientific, technical/technological, legislative, cost and market gaps or barriers. An overall recommendation was then proposed on the basis of the average score for each sector (favorable/partially favorable/unfavorable conditions for rapid sector and market development). An unfavorable rating may indicate a current limitation to enter or develop a sector, but also opportunities if gaps and barriers can be overcome. Conversely, a favorable rating may indicate a sector of activity that is easy to integrate but where a significant competition in the market may already exist. The rating scale for each element was based on the following criteria (Table 2.)

## Figures and Tables

**Table 1:** SWOT analysis of microalgae research, development and marketing in the European Atlantic Area in 2020.

Strengths	Weaknesses	Opportunities	Threats
Limitation of freshwater consumption No competition with agricultural lands Recycling of water, nutrients and energy Organic label and eco-conception Excellence of R&D and/or availability of trained workforce A wide range of stakeholders Quality certification and labels Traceability of products Wide range of molecules and biodiversity, some of them specific to microalgae Confinable productions Coastal ratio of Atlantic Area and long experience/know how in aquaculture practices	Competition with mature markets (crops, terrestrial plants) and similar cheaper products (e.g., synthetic carotenoids, fish oils) Prohibition to sell some species and related products in non-EU markets (e.g., China) Lack of uniformity in enacting legislation (including planning and building regulations and start-up incentives) between countries in the EU Administrative processes for novel food authorization Cost to approve the marketing of microalgae-related products (e.g., meeting regulatory/legislative demands) Low number of species that are industrially grown in regard to the biodiversity Production scale of existing growth systems Lack of information demonstrating reproducible bulk production levels Lack of scientific demonstration of the biological activity of microalgae-related products Sustainability of long-term continuous production Technological transfer from labs and R&D platforms to the industrial sector High cost of operation facilities Insufficient supporting investment in consortia of industry and academia Insufficient public funding of R&D Lack of information and knowledge about potential consumers and low acceptability of consumers for microalgae and related products. Still not truly a “major” societal concern for consumers + lack of wide/large-scale studies with robust scientific data on human nutrition Lack of information and knowledge about microalgae companies towards consumers (social networks) Weakness of the sector consortium labels, lobbying, visibility, compared to more mature branches	Cultivation conditions and methods to improve the ratio added value product/by-products production Harvest improvements (centrifugation, filtration, flocculation, innovative methods, cost-effective technological breakthrough) Circular workflows within industrial activities (nutrient recycling systems, syngas, CO2 sequestration, anaerobic or aerobic digestion of microalgae biomass) Cosmetics (moisturing and texturing polysaccharides, bioactive lipopeptides) Replacement of antibiotics by microalgae and derivatives, development of new antibiotics and photosensitizers Pigments for food, nutraceuticals and pharmaceuticals (carotenes, xanthophylls, phycobiliproteins, mycosporin-amino-acids, polyphenols, others) Microalgal-based ingredients and feedstuffs Light/electronic design and allied energy/nutrient management Natural microalgal species selection and non-GMO improvement GM microalgae for health: production of complex eukaryotic molecules (glycosylated proteins) Biorefinery approach leading to economical resiliency	Low financial investment to stimulate innovation and risk taking, especially for maturation funds Competition with other markets, regions, countries and low-cost producers, high labor costs Low financial guarantees to secure innovation risks Difficult transfer of technology for industrial upscaling of R&D discoveries Lack of labels and good practices, standards towards productions practices Fast saturation of niche markets (e.g., astaxanthin)

**Table 2:** Criteria for the traffic light analysis of microalgae activity sectors in the European Atlantic Area, according to scientific, technical/technological, legislative, cost and market gaps and barriers.

Gaps and Barriers	Green Light	Orange Light	Red Light
Scientific	Scientific research is advanced and robust scientific data exist to develop the sector and go to markets	Scientific gaps exist for the development of the sector but knowledge is sufficiently advanced to envisage industrial and market developments	Major lack of scientific knowledge limiting the possibility and profitability of industrial developments
Technical/Technological	The technology makes it possible to obtain the product or service in an easy and reproducible way	Technological developments are still needed to ensure the production of the product or service at industrial scale	There are technological barriers that block the production of the good or service
Legislative	No legislative constraint to integrate the sector and the market	Legislation limits the development of the product or service and market accessibility	The legislation is very restrictive and strongly limits or forbids the development of the sector or product
Cost	Low to moderate costs and investments to integrate the sector and the market	Mild to high cost to integrate the sector but the market is profitable	The costs for the development of the sector or product are too high for a current profitability and market development
Market	The market is open, favorable and expanding. Competition remains limited by a high demand or a low number of competitors.	The market is open and favorable, but there is a lot of competition, or the market is still confidential	There is no market for this product or service, or it is a niche market. The current added value does not stimulate investments for market development.

## Supplementary Materials

The following are available online at https://www.mdpi.com/article/10.3390/md19060319/s1, Table S1: Detailed traffic light analysis of the main scientific, technical, legislative, development cost and market gaps and barriers, and overall assessment of near-future market opportunities for all microalgae sectors. Figure S1: Survey questionnaire.
