# Supplementary material for: Improving Microalgae Research and Marketing in the European Atlantic Area: Analysis of Major Gaps and Barriers Limiting Sector Development
Source: Mar Drugs. 2021 May 30;19(6):319. doi: 10.3390/md19060319 (PMC8229015; doi:10.3390/md19060319)
Supplement: Supplementary file 1 [file marinedrugs-19-00319-s001.zip › marinedrugs-1185948-SI.pdf]

Supplementary Information

Table S1: Detailed traffic-lights analysis of the main scientific, technical, legislative, development cost and market gaps and barriers, and overall assessment of near future market opportunities for all microalgae sectors.

**Sector**	**Application**	**Scientific Gap and barrier**	**Technical Gap and barrier**	**Legislative Gap and barrier**	**Cost Gap and barrier**	**Market Gap and Barrier**	**Global**
Food	Pigments	🟢	🟢	🟢	🟡	🟢	🟢
	Proteins including Phycobiliproteins	🟢	🟢	🟢	🟡	🟢	🟢
	Omega-3	🟢	🟢	🟢	🟢	🟢	🟢
	Antioxidants	🟢	🟢	🟢	🟢	🟢	🟢
	Fitness, Bodybuilding and weight loss	🟡	🟢	🟢	🟢	🟢	🟢

Feed	Pigments	🟢	🟢	🟢	🟡	🟢	🟢
	Proteins	🟢	🟢	🟢	🟢	🟢	🟢
	Omega-3	🟢	🟢	🟢	🟢	🟢	🟢
	Antioxidants	🟢	🟢	🟢	🟢	🟢	🟢

**Sector**	**Application**	**Scientific Gap and barrier**	**Technical Gap and barrier**	**Legislative Gap and barrier**	**Cost Gap and barrier**	**Market Gap and Barrier**	**Global**
Biomaterials / Bioplastics	Polyhydroxyalkanoates (PHA)	🟡	🟡	🟢	🔴	🟡	🟡
	Biotissues and xenograft of microalgae-based matrices for tissular engineering [32–35]	🟡	🔴	🟢	🔴	🔴	🔴
	Polylactic acids	🟢	🟡	🟢	🔴	🟢	🟡
	Bio-polyethylene	🟡	🟡	🟢	🔴	🟢	🟡
	Bio-PVC	🟡	🟡	🟢	🔴	🟢	🟡
	Biofilters	🟢	🟢	🟢	🟢	🟢	🟢

Sector	Application	Scientific Gap and barrier	Technical Gap and barrier	Legislative Gap and barrier	Cost Gap and barrier	Market Gap and Barrier	Global
Human, and animal health	Malnutrition, food protein supply	🟢	🟢	🟢	🟡	🟢	🟢
	Vaccines, Recombinant proteins *	🟡	🟡	🟡	🔴	🟢	🟡
	Cicatrization	🟡	🟢	🟢	🟢	🟢	🟢
	Antioxidant	🟢	🟢	🟢	🟢	🟢	🟢
	Metabolic syndrome (Obesity/Diabetes)	🟡	🟡	🟢	🔴	🟢	🟡
	Cancer prevention	🟢	🟡	🟡	🔴	🟢	🟡
	Cancer treatment and potentiation of drugs	🟡	🟡	🔴	🔴	🔴	🔴
	Lutein/Zeaxanthin for DMLA and Cataract	🟢	🟡	🟢	🟡	🟢	🟡
	Fertility improvement						

	Alternative to antibiotics in animal breedings	🔴	🔴	🔴	🔴	🟡	🔴
	Antidepressant and anxiolytic (PUFAs)	🟢	🟢	🟢	🟡	🟢	🟢
	Cardioprotection (PUFAs)	🟢	🟢	🟡	🟡	🟢	🟢
	Anti-inflammatory	🟢	🟢	🟡	🟡	🟢	🟢
	Normoglycemiant	🟢	🟢	🟢	🟢	🟡	🟢
	Immunity stimulation (PUFAs)	🟡	🟢	🟡	🟢	🟢	🟡
	Microalgae toxin standards for pharmacology and environmental diagnostic	🟢	🟢	🟡	🟡	🟢	🟢
	Neuroprotection	🔴	🔴	🟡	🔴	🟡	🟡

	Hepatoprotection	🟢	🟢	🟡	🟢	🟢	🟢
	Limitations of Pregnancy complications (PUFAs)	🟢	🟢	🟡	🟢	🟢	🟢
	Prevention and treatment of viral, bacterial and fungal infections	🟢	🟢	🟡	🟡	🟢	🟡
	Blood circulation	🟡	🟢	🟡	🟡	🟢	🟡
	Coagulation	🔴	🔴	🔴	🔴	🟡	🔴
	Fluorescent pigments for diagnostic and prognostic	🟢	🟡	🔴	🟡	🟡	🟡
	Tumor and antibacterial Photosensitizers	🟢	🟢	🟢	🟡	🟢	🟢

		🟡	🔴	🟢	🔴	🟢	🟡
Biofuels	Biodiesel	🟢	🟡	🟢	🔴	🟢	🟡
	Biomethane	🟢	🟡	🟢	🔴	🟢	🟡
	Bioethanol/biobutanol	🟢	🟡	🟢	🔴	🟢	🟡
	Biohydrogen	🔴	🔴	🟡	🔴	🟢	🟡
	Biokerosene	🔴	🔴	🟡	🔴	🟢	🟡
Cosmetics and Nutraceuticals	Proteins	🟢	🟢	🟢	🟢	🟢	🟢
	Polysaccharides/EPS	🟢	🟢	🟢	🟢	🟢	🟢
	PUFAs	🟢	🟢	🟢	🟡	🟢	🟢

	Pigments including Phycobiliproteins	🟢	🟢	🟢	🟡	🟢	🟢
	Polyphenols	🟡	🟡	🟢	🟡	🟢	🟢
	Minerals	🟢	🟢	🟢	🟢	🔴	🟡
	Vitamins	🟡	🟡	🟢	🔴	🟡	🟡
	Antioxidant	🟢	🟢	🟢	🟢	🟢	🟢
	Anti-aging	🟢	🟢	🟢	🟢	🟢	🟢
	Photoprotection (including MAA and scytonemin)	🟡	🟡	🟢	🔴	🟡	🟡
	Anti-allergenic	🔴	🔴	🔴	🔴	🟡	🔴

	Thickeners	🔴	🔴	🟢	🔴	🟡	🔴
	Moisturing	🟡	🟡	🟢	🟡	🟡	🟡
	Thalassotherapy and microalgae from thermal water	🟡	🟢	🟢	🟢	🟢	🟢

* Including hormones, cytokines, proteic/peptidic/oligosaccharidic vaccinal antigens produced by GMO-strains.

Figure S1. Survey questionnaire.

High value added industrial opportunities for microalgae in Europe

Survey

EnhanceMicroAlgae is a project funded by the European INTERREG Atlantic Area program that aims to facilitate the transfer of knowledge and technology between academic experts and companies in the microalgae sector. The aim of the project is to boost the industrial exploitation of microalgae and the development of high value added markets.

We have identified you as an essential expert in the field and are seeking your help in participating in a short survey (30 questions) designed to gather information about the technological, market, legislative and social gaps and barriers that you view as limiting the development of the microalgae sector.

The data you provide will be used to identify strengths, weaknesses, threats and opportunities for the development of high value added microalgae products and markets.

Survey responses will be anonymized and only if you provide contact details may you be contacted with any follow-up questions. The responses will be analyzed respecting the regulation of the European parliament of the council on the protection of individuals with regard to processing personal data. No personal name or data will be presented in the conclusions and diffusions of this survey. For more information on the EnhanceMicroAlgae privacy policy, please contact us.

The summarized, anonymized results of the survey will be made available via this website: www.enhancemicroalgae.eu.
In addition to completing the survey, we would be grateful if you could help us reach additional respondents by forwarding this survey to other colleagues and organizations that you think may have an interest in commercial exploitation of microalgae.

Personal, Institute & Company Data

1- What is your personal name?

□ I prefer not to give this information

2- What is the name of your institution/company?

□ I prefer not to give this information

3- Where is your institution/company located (city and country)?
Precise if the algal-production base is different to the headquarters.

4- Please select your organization type.
□ Academic/university/faculty
□ Public funded non profit institute
□ Private company
□ Other (please detail):

5- What are your sectors of activity? (please indicate all that apply)

□ R&D science

□ Equipment conception, design and manufacture
□ Microalgae cultivation and biomass production
□ Transformation (Extraction-purification-biorefinery)
□ Analysis platform
□ Advice-project management
□ Manufacturer of products based on microalgae ingredients
□ Biobank

6- What are your domains of activity/business? (please indicate all that apply)

□ Human food / Nutraceutical / Health food
□ Aquaculture or farming feed stuffs
□ Bioenergy
□ Cosmetics
□ Pharmaceuticals
□ Chemicals
□ Environment
□ Biotechnology
□ Equipment - software
□ Advice – project management
□ Other (please precise):

7- What is the size of your institution/company?

□ <5 employees
□ 5-10
□ 10-50
□ >50

8- How long has your institution/company been in activity within the microalgae sector?
□ <2 years
□ 2-5
□ 5-10
□ >10

9- What was the financial turnover of your institution/company last year? (€; please assume €=US$=£)
□ <50 000
□ 50 000- 100 000
□ 100 000 – 500 000
□ 500 000 – 1 000 000
□ > 1 000 000
□ I don't know or prefer not to give this information

GAPS & TECHNOLOGICAL BARRIERS (CURRENT LIMITATIONS AND WEAKNESSES)

10- Classify the following barriers to the development of high value added microalgae related products, from most important (n°1) to less important (n°5).

- Scientific barrier (research and development) :
- Technical/technological barrier (equipment and production capacity, purification technology):
- Legislative barrier (regulation, such as use of sewage nutrients for food-microalgae):
- Economic barrier (market):
- Socio-cultural barrier (consumer acceptability):

11- In your opinion, how important are the following scientific barriers in limiting the industrial development of microalgae and related products in the European Atlantic Arc? (crucial, very important, less important)

- Lack of innovation and redundancy of studied products and species

- Insufficient public funding of R/D
- Insufficient supporting investment in consortia of industry and academia
- Lack of genetic and ecophysiological data on microalgae species
- Lack of scientific demonstration of the biological activity of microalgae related products
- Lack of international coordination of scientific teams
- Other (please detail):

12- In your opinion, how important are the following aspects limiting industrial developments in support of microalgae and related products in the European Atlantic Arc. (crucial, very important, less important)

- Access to technical/technological information about strains, culture, equipment and products
- Production scale of existing growth systems
- Research of heterotrophic algal production systems
- Lack of information demonstrating reproducible bulk production levels
- Development of microalgae harvesting systems
- Development of commercial-scale systems for the extraction and purification of metabolites
- Energetic yield
- Sustainability of long-term continuous production
- Technological transfer from labs and R/D platforms to the industrial sector
- Other (please detail):

13- Independently from their importance for the sector, how do you estimate the possible progression of the following technical aspects in the next 10 years? (slow progress, medium progress, fast progress)

- Micro-algal species selection
- Biomass production
- Harvest
- Extraction
- Scale-up of production systems
- Fractionation and purification
- Photobioreactor design
- Contamination/ predator invasion/ weed-algae invasion
- Quality control monitoring and certification (to meet regulations)
- Light/electronic design and allied energy/nutrient management
- Other (please detail):

14- Please indicate the importance of the following legislative barriers limiting the industrial development of microalgae and related products. (crucial, very important, less important)

- Difficult access to legislative information and updates for microalgae
- Low number of species that can be grown
- Prohibition to sell some species and related products in non-EU markets (e.g. China)
- Nagoya protocol on access and benefit sharing, limiting the exploitation of non-native species and intelectual property
- Complexity and constraints related to EU regulation on genetically modified strains
- Lack of uniformality in enacting legislation (including planning and building regulations, start-up incentives etc) between countries in the EU
- Administrative constraints to create a company
- Commercialization from non-EU nations (e.g. China, US) of microalgae products marketed as dietary supplements
- Other (please detail):

15- Please indicate the importance of the following economic barriers limiting the industrial development of microalgae and related products

- Competition with low-cost producers outside from your geographic zone
- Competition with mature markets (crops, terrestrial plants)
- Cost to approve the marketing of microalgae-related products (e.g., meeting regulatory/legislative demands)
- Insufficient analysis of the market in the Atlantic Arc to identify new perspectives
- Low short-term profitability of high-added microalgae products

- Low economic support to the sector
- High cost of operation facilities
- Other (please detail):

16- Please rank the following socio-cultural barriers limiting the industrial development of microalgae and related products (crucial, very important, less important)

- Insufficient training/qualification of workforce for microalgae industries
- Low acceptability of consumers for microalgae and related products
- Lack of information about potential consumers
- Fear that microalgae are contaminated by pollutants, heavy metals and toxins
- Validity and reliability of sources of information for consumers (specifically dedicated to microalgae)
- Lack of visibility and communication of microalgae companies towards consumers (social networks)
- Other (please detail):

17- In your opinion, is there a market opportunity for products obtained from GM- microalgae, and if so, indicate in which sector and the forecast application?

- Yes or no:

- Please select the sector and indicate the applications you forecast:

□ Human food / Nutraceutical / Health food
□ Aquaculture or farming feed stuffs
□ Bioenergy
□ Cosmetics
□ Pharmaceuticals
□ Chemicals
□ Environment (e.g. bioremediation)
□ Biotechnology
□ Equipment - software
□ Advice – project management
□ Other (please precise):

THREATS (FUTURE RISKS): market, economy, technique

18- The following proposals lists some potential threats that could limit the future development of the microalgae industrial sector in your geographic area. Can you identify the ones that seem the most important in your opinion and propose other limitations or threats that you believe as relevant for the next 10 years?
□ Competition with other markets, regions, countries and low-cost producers, high labour costs
□ Minor market for very innovative products and services
□ Access limitation to production sites located with ready access to the seashore
□ Low financial guarantees to secure innovation risks
□ Low financial investment to stimulate innovation and risk taking
□ Sub-optimal climate (weather) to grow microalgae in open pond-systems
□ Low maturity, with allied risks, of the sector
□ Lack of consortia of academic and industrials
□ Difficult transfer of technology for industrial upscaling of R/D discoveries
□ Risk of data and intellectual property leakage
□ Other (please detail):

19- In your opinion, what is the main factor explaining the failure of SMEs or spin-off exploiting high-added value molecules obtained from microalgae in Europe?
Indicate some possible solutions to avoid this failure:

STRENGTHS & OPPORTUNITIES (FOR THE FUTURE DEVELOPMENT)
20- Among the following proposals, which ones seem the most relevant to positively differentiate the geographic area in which your company operates from other European areas ? (highly relevant, relevant, irrelevant)

- Geographic origin
- Access to coastal areas and seawater
- Quality certification and labels

- Traceability
- Excellence of R/D and/or availability of trained workforce
- Creation of an Interregional database updating microalgae producers, transformers and research projects in your geographic area
- Creation of an international cluster federating all stakeholders
- Development of experimental platforms for technology transfer
- Other (please detail):

21- What is the contribution of your geographic area to the following world microalgae markets? (0, 10, 25, 50, 100%)

- Human food / Nutraceutical / Health food
- Aquaculture or farming feed stuffs
- Bioenergy
- Cosmetics
- Pharmaceuticals
- Chemicals
- Environment
- Biotechnology
- Equipment - software
- Advice – project management
- Other (please precise):

22- What percentage of growth do you foresee the following (global) microalgae markets in the next 10 years? (0, +10, +50, +100, >100%)

- Food
- Nutraceutical
- Feed
- Bioenergy
- Cosmetics
- Medical
- Chemical
- Environment
- Aquaculture
- Biotechnology
- Agriculture
- Other (please precise):

23- Please indicate how promising you see the following markets using microalgae in the next 10 years? (very promising, moderately promising, not promising)

- Development of kits to detect microalgae toxins in the environment
- Microalgae-based vaccines and hormones (recombinant proteins, new drugs)
- Cosmetics (moisturing and texturing polysaccharides, bioactive lipopeptides)
- Design and develoment of photobioreactors, growth and harvesting systems
- Replacement of antibiotics by microalgae and derivatives in aquaculture
- Replacement of fertilisers, pesticides, antifungals and antifouling by microalgae and derivatives
- Bioenergy (biohydrogen, bioethanol, CO2 captation, biomethane and biofuel, solar energy)
- Bioplastics and biomaterials (silica, resins, waxes and polysaccharides)
- Pigments for food, nutraceuticals and pharmaceuticals (carotenes, xanthophylls, phycobiliproteins, mycosporin-amino-acids, polyphenols, others)
- PUFAs for food and health applications
- Polysaccharides for health applications (cicatrisation, anticoagulant, antithrombotic, antitumor, antivenom, tissular engineering, antiviral, hygiene)
- Adjuvants for green chemistry (bioethanol, biosolvents, aromas and perfumes, essential oils)
- Renewable food resources for long-term trips
- Analytical chemistry compounds (toxins) used as standards
- Other (please detail):

24- In your opinion, which R/D developments and downstream processes technologies will have the greatest impacts enhancing the commercial microalgae sector in the next 10 years? (very impactful, moderately impactful, not impactful)

- Cultivation conditions and methods to improve the ratio added-value product/by-products production
- More efficient strain screening for desired product
- Harvest improvements (centrifugation, filtration, flocculation, innovative methods)
- Extraction techniques (supercritical CO_2, PLE, MAE, UAE, PEF, membrane-based separations)
- Extraction solvents (organics, ionic liquids, surfactants thermodynamics studies)
- Purification (Chromatographic techniques)
- Circular workflows within industrial activities (nutrient recycling systems, syngas, CO2 sequestration, anaerobic or aerobic digestion of microalgae biomass)
- Development of bioassay guided techniques for new drugs discovery
- New biocatalytic pathways for production of desired compounds
- Others (please detail):

25- Which of the following arguments do you see as having a positive marketing impact and add financial value to a microalgae-based product? (highly positive, moderately positive, neutral)

- Limitation of freshwater consumption
- No competition with agricultural lands
- Biodegradability of products
- Recycling of water, nutrients and energy
- Biorefining and by-products valorization
- Organic label and eco-conception
- Vegan label and eco-consumption
- Traceability of products
- Geographic zone label of product (e.g. Marine Atlantic, local, European)
- Biological activity certificate
- Originality of the resource
- Other (please precise):

SOME BETS FOR FUTURE

26- In your opinion, what will be the major breakthrough in algal research for nutraceutical applications within 10 years?

27- In your opinion, what will be the major breakthrough in algal research for human health/ pharmaceutical applications within 10 years?

28- In your domain of expertise, which innovative products based on microalgae would you like to be marketed in the 10 next years?

29- Do you think that there is a lack of specific skills or formations for scientists dealing with legislative and marketing issues in the microalgae sector?

30- Which key factors are critical for the long-term viability of a microalgae company?
